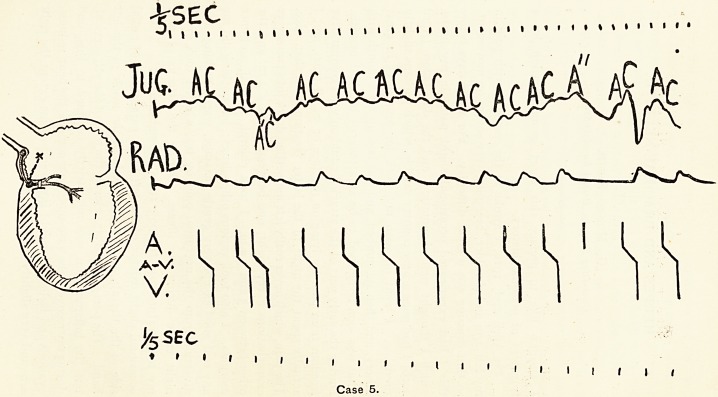# Cardiac Arrhythmia: Five Cases

**Published:** 1911-12

**Authors:** Carey Coombs

**Affiliations:** Assistant Physician, Bristol General Hospital


### CARDIAC ARRHYTHMIA: FIVE CASES.

BY

Carey Coombs, M.D., M.R.C.P. Lond.,

*Assistant Physician, Bristol General Hospital.*

---

These five cases are selected, not because of any new data brought to light by a study of them, but because they will serve as a means for demonstrating the principal facts on which the modern conceptions of cardiac arrhythmia are based.

First, certain facts as to the anatomy and physiology of the cardiac muscle must be recalled. The primitive heart is a muscular tube, the powerfully contracting chamber is a later development, and the valves and pericardium are convenient appendages added later still. Traces of this tube are still to be found in the mammalian heart. These are (*a*) the sino-auricular node, situated at the junction of the sinus venosus, the opening of the superior vena cava, with the right auricle; (*b*) the auriculo-ventricular node, situated in the inter-auricular septum, just above the pars membranacea of the inter-ventricular septum; (*c*) the auriculo-ventricular bundle, first described by Professor Stanley Kent, which runs from the node into the inter-ventricular septum, and splits into two branches, one for each ventricle, each breaking up again and again for distribution throughout the ventricular musculature. No definite separate muscular connection has yet been proved between the two nodes, which, it must be remembered, are the main points of junction between the nerves and the musculature of the heart.

So much for anatomy. Next it is necessary to state the five separate functions of the cardiac muscle. (1) Rhythmicity, or the power of originating within itself the necessary stimulus at regular intervals. (2) Excitability, or the faculty of being impressed by that stimulus into a response. (3) Conductivity, or the power of carrying the stimulus from one part of the heart to another. (4) Contractility, or the power of contraction in response to stimulation, such as is common to all kinds of muscle. (5) Tonus, or the property by which the muscular chambers are prevented from complete relaxation even in full diastole. The stimulus begins normally at the sino-auricular node, passing thence through the auricular walls to the auriculo-ventricular node, and onwards into the ventricular walls by way of the auriculo-ventricular bundle and its branches. These functions, though essentially muscular, are subject to exaltation or depression by nervous influences transmitted through the vagus and sympathetic.

By means of the graphic methods of studying the cardiac movements we have not only arrived at a definite understanding of the nature of the different types of arrhythmia, but we have also found it possible to analyse with tolerable certainty the causative factors in individual cases of irregularity. The graphic methods in use are of two principal kinds—those that record the movements of the heart or blood-vessels, and those that record the changes in the electrical potential of the cardiac walls. The latter study is a very intricate one, but it has already yielded most important data. It is, however, not freely applicable to clinical medicine, and the cases before you to-night have been investigated entirely by records taken direct from two parts of the circulation, the radial artery representing ventricular and the internal jugular vein representing auricular movements. The tracings here shown are copies from originals taken with Mackenzie's ink polygraph. The points to remember in their interpretation are these: (1) The time-relation of the waves is the thing that matters; their amplitude may be ignored. (2) The radial tracing is taken because it is a convenient index of the time at which one definite event in the cardiac cycle, namely ventricular systole, has occurred. (3) The first step is to measure on the radial tracing the distance from the ordinate line with which the tracing begins or ends to the beginning of the upstrokes which indicate the pulse waves. (4) Next these measurements are transferred to the jugular

Case I.

Case 2.

tracing by means of callipers or compasses. For instance, having measured the distance X—Y in the first tracing (Fig. 1), we transfer this measurement to the jugular tracing, and find the position of Y[1] by fixing X[1] at the point of origin of the tracing. (5) Allowing for the difference in time (0.1 sec.) between the carotid and radial pulses, we find waves in the jugular tracing corresponding in time with those of the radial tracing. These are the carotid or " C " waves, the upstrokes of which correspond with the beginning of ventricular systoles. (6) The wave preceding each carotid, ventriculo-systolic wave is the " A " wave, auriculo-systolic in origin. (7) The waves marked " V " may be ignored so far as this paper is concerned. They correspond with the onset of ventricular diastole.

At the side of each tracing I have drawn a schematic heart, in the first of which 1=the sino-auricular node, 2=the auriculo-ventricular node, and 3=the auriculo-ventricular bundle. In each case points of abnormal stimulus production are indicated by crosses, " X," paths of such abnormally produced stimuli by dotted lines, interruption of conductivity by a gap in the line depicting the a-v bundle, and failing contractility by shading of the injured portion of myocardium. Beneath each tracing there is a diagram to show the sequence of the chief events in the cardiac cycle: AUR=auricular systole, A—V=auriculo-ventricular stimulus condition, and VENT.=ventricular systole. The line at the top marks off fifths of seconds.

**Case 1** is that of a neurotic girl. When I saw her first her pulse rate was 40 per minute, and as there were cardiac symptoms, but no physical signs of disease, I determined to take a tracing. When this was done the pulse beat 80 per minute, and regularly except for a short period of tachycardia, seen in the second half of the tracing printed here. The waves on the jugular curve which corresponded with the tachycardial radial waves are marked " C[1]." Each of these is preceded at the normal interval ($\frac{1}{5}$ sec.) by an " A " wave. Auricle and ventricle are therefore beating in normal sequence, though at a quickened rate. The stimulus is therefore being originated at the normal spot, the sino-auricular node; the abnormally quickened rate is probably due to depression of vagus control. This type is known as " sinus irregularity."

**Case 2** is of some little interest. It is that of a girl of ten years or so, with rheumatic carditis, who has been under my observation for about two years. During that time her pulse has almost always been subject to premature beats (known as "extrasystoles"), occurring at varying intervals. One of these, marked "R 1," is shown in the tracing (Fig. 2). The records show that the premature ventricular beat is preceded at the normal distance by an auricular beat; a long strip of tracing showed also that the distance between each premature beat and the preceding normal beat was constant. From this we may deduce that (*a*) the site of production of such premature beats is in the auricle, and (*b*) such beats all arise from some one special site. It is of course possible to argue that the site is none other than the sino-auricular node, and that the premature beat is due to a recurrent disturbance of innervation; but I think it is unlikely that a nervous disturbance would continue to produce isolated extrasystoles, all of the same type, month after month and year after year. It is much more likely that there is in the auricular wall some irritable spot, at which stimulus material is being built up too fast, or susceptibility to stimulation abnormally keen. We can only guess at the nature of such a focus. In the active phases of cardiac rheumatism two varieties of focal lesion are to be found in the myocardium, one constant and the other occasional. The constant lesion is the submiliary nodule, described fully in another paper,[1] the rarer one is obliterative arteritis. For two reasons I am inclined to attribute this extrasystole to the arterial lesion. First, extrasystoles are commoner in the arterio-sclerotic heart than in any other form of organic cardiopathy; second, they appear in less than 5 per cent. of all cases of the cardiac rheumatism of childhood, and cannot therefore be associated with a lesion, which, like the submiliary nodule, is always present.

**Case 3** (Fig. 3) is that of a man aged 27 with mitral stenosis. His lips were blue, his feet swollen, and his cardiac reserve very limited, for he could scarcely walk even on level ground without distressing breathlessness. Roughly examined with the finger, his pulse seemed almost regular. A tracing, however, brings out two points. First, the pulse is totally irregular, *i.e.* no two consecutive beats are of the same length; second, the auriculo-systolic wave has quite disappeared from the jugular tracing, leaving only waves of great amplitude, ventriculo-systolic in time. This is the condition known as "pulsus irregularis perpetuus" (Hering), or better still, "total arrhythmia." The original theory of "nodal rhythm" provisionally offered by Mackenzie has been withdrawn, and this irregular pulse is now

[1] *J. Path. and Bacteriol.*, 1911, xv. 48.

Case 3.

Case 4.

accounted for, according to the researches of Lewis[1] and others, by the process known as "auricular fibrillation." This process is depicted in a rather crude way in the schematic drawings. The suggestion is that the auricle, failing in the impossible task it has been set by the constriction of the mitral valve, has abandoned its normal co-ordinate systole, which is replaced by numerous disconnected, ineffective, partial twitchings of the auricular wall. Each part of the auricular wall has become a "law unto himself," and generates impulses which are pelted in a confused series into the ventricle through the conducting bundle, producing a haphazard sequence of ventricular responses. It is this condition which is the one great indication for the use of digitalis, according to Mackenzie.

**Case 4** was a very interesting one from the diagnostic and prognostic point of view. The patient, an elderly man, had very sclerotic arteries and an extraordinarily irregular pulse, as the tracing (Fig. 4) shows. He suffered no great distress, however, apart from uncomfortable sensations over the heart. The pulse was so irregular that at first I thought it must be an example of the total irregularity exemplified in Case 3. The tracing, however, showed that whatever the arrhythmia was due to it was certainly not of the type associated with auricular fibrillation, for it presented distinct evidences of efficient auricular systole. I have interpreted the tracing as a jumble of extrasystoles, with a minor degree of heart-block due to digitalis. I do not, however, wish to press this explanation, but only to emphasise the fact that by means of the tracing we were enabled to exclude the serious prognostic element introduced by a diagnosis of total arrhythmia. The man was in the General Hospital under Dr. Parker, who took a great interest in the case, and kindly allowed me to take tracings. He was allowed to get up and go about the ward, and went out quite comfortable in spite of his arrhythmia. I have seen several such cases of multiple extrasystoles simulating "total irregularity."

**Case 5** was of a very different order. A stout alcoholic, of about 40 years, came into the Hospital urgently ill, almost moribund, in fact. He was intensely dyspnœic, and œdematous as high as his chest. Under treatment he improved remarkably, but was unable to resume work. The tracing, part of which appears in Fig. 5, was taken some weeks after he had left the wards. It shows three abnormalities. (1) Early in the tracing an auricular extrasystole is seen. (2) Throughout the tracing the radial pulse shows true alternation, *i.e.* the beats are of equal length but alternate in amplitude,

[1] *Heart*, 1909–10, i. 306.

a small beat being always interposed between two large beats. This alternation is always a grave sign, since it proves exhaustion of contractility. The contractile substance is built up with difficulty in such hearts ; a full ventricular systole so exhausts the stock that the normal diastolic pause does not suffice for its replenishment, and the next beat is therefore a small one. This gives the heart a more adequate rest ; time is allowed for a better storing of contractile substance, which once more permits of, and is exhausted by, a full beat, and so on. (3) Towards the end of the tracing a radial beat is missing. Reference to the jugular tracing shows that this was due not to failure of the auricular systole, which took place normally ("$A^{11}$"), but to lack of the normal ventricular sequence : that is to say, the impulse which duly stimulated the auricle to contraction was "blocked" in its passage from auricle to ventricle through the bundle of His, as the schematic heart shows. Thus the tracing showed (*a*) an abnormally irritable spot in the auricle, (*b*) exhaustion of ventricular contractility, (*c*) some impairment of the conducting function. The man died rather suddenly, about three weeks after this tracing was taken.

I do not think it fair to argue that a polygraphic examination is of value in every case of heart disease ; indeed, it is possible to arrive at an adequate comprehension of most cases presenting cardiac irregularities without one, provided that the principles underlying the origin of arrhythmia are understood. Nevertheless, there are many cases in which tracings give information of vital importance, which cannot be arrived at in any other way. It is scarcely necessary to add that the use of the polygraph gives the individual practitioner, as it has already given the profession as a whole, a safe basis for action in cases of cardiac arrhythmia, instead of the harassing uncertainty to which we are subject unless armed with a knowledge of facts.

Case 5.

## Figures and Tables

**Case 1. f1:**
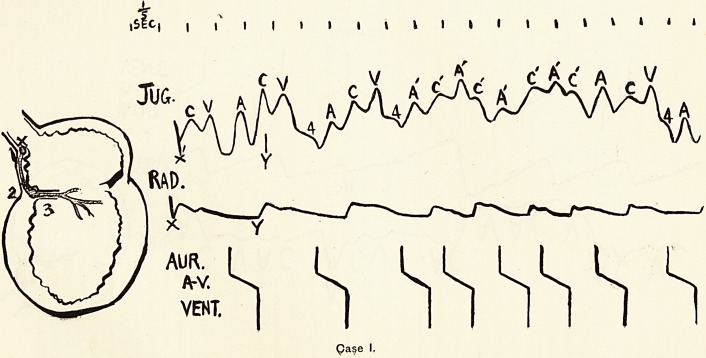


**Case 2. f2:**
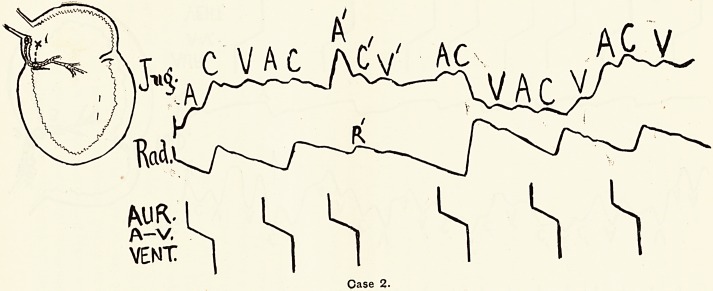


**Case 3. f3:**
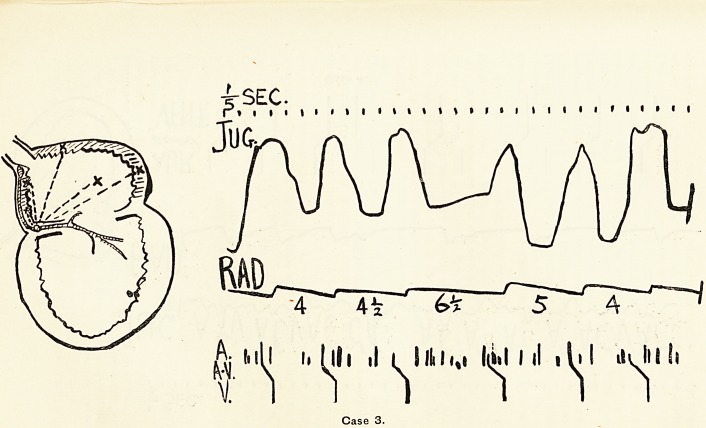


**Case 4. f4:**
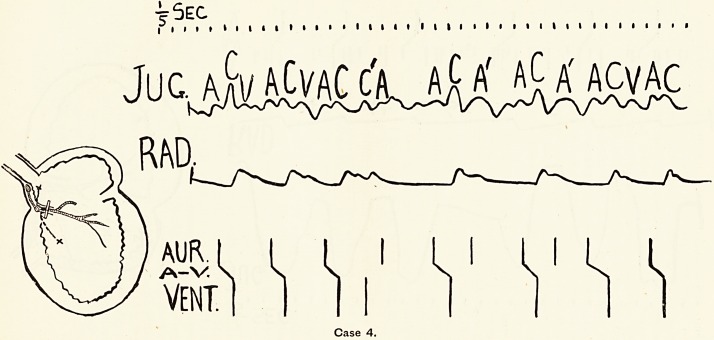


**Case 5. f5:**